# Reference intervals of biochemical parameters in Chilean adults

**DOI:** 10.5937/jomb0-44156

**Published:** 2024-01-25

**Authors:** Pablo Letelier, Rodban Acuña, Ignacio Garrido, Jorge López, Guillermo Sanhueza, Caren Seguel, Ismael Riquelme, Neftalí Guzmán, Alfonso H. Hernández

**Affiliations:** 1 Universidad Católica de Temuco, Facultad de Ciencias de la Salud, Departamento de Procesos Diagnósticos y Evaluación, Laboratorio de investigación en Salud de Precisión, Chile; 2 Universidad Autónoma de Chile, Faculty of Health Sciences, Institute of Biomedical Sciences, Chile

**Keywords:** references intervals, biochemical markers, interindividual variability, referentni intervali, biohemijski markeri, interindividualna varijabilnost

## Abstract

**Background:**

Establishing reference intervals (RIs) in clinical laboratories is essential, as these can vary due to inter-individual variability as well as the analytical methods used. The purpose of this study was to determine RIs for markers and ratios biochemical in apparently healthy Chilean adults.

**Methods:**

A sample of 1,143 data was selected from the Universidad Católica de Temuco, Clinical Laboratory database, La Araucanía Region, Chile, which were analysed by sex. The Tukey's Fences was used to detect outliers and the RIs were established using the non-parametric method.

## Abstract (sr)

**Uvod:**

Uspostavljanje referentnih intervala (RI) u kliničkim laboratorijama je od suštinskog značaja, jer oni mogu varirati zbog varijabilnosti među pojedincima, kao i zbog korišćenih analitičkih metoda. Svrha ove studije je bila da se odredi RI za markere i biohemijske odnose kod naizgled zdravih odraslih Čileanaca.

**Metode:**

Uzorak od 1.143 podataka odabran je iz baze podataka Universidad Católica de Temuco, kliničke laboratorije, La Araucanía Region, Čile, koji su analizirani prema polu. Tukeižs Fences je korišćen za otkrivanje odstupanja, a RI su uspostavljeni korišćenjem neparametarske metode.

**Rezultati:**

Dobijeni RI za ženski pol su: urea 2,48-7,35 mmol/L, AST 0,20-0,69 mkat/L, ALT 0,13-1,12 mkat/L, alkalna fosfataza 0,83-2,5 mkat/L, ukupni bilirubin 3,3 l-7 mkat/L L, direktni bilirubin 1,5-4,9 mmol/L, mokraćna kiselina 148-362 mmol/L i ukupni proteini 66,3-80,3 g/L i za muškarce: urea 3,28-8,17 mmol/L, AST 0,22-0,8, ALT 0,18-1,9 mkat/L, alkalna fosfataza 0,93-2,33 mkat/L, ukupan bilirubin 4,58-24,0 mmol/L, direktni bilirubin 1,9-7,5 mmol/L, mokraćna kiselina 202-458 mmol/L i ukupni proteini 67,4-83 g/L. FIB-4 i APRI indeksi su predstav ljali raspon od 0,2-1,6 i 0,12-0,5 (u oba pola), a AST/ALT indeks je bio 0,5-2 kod žena i 0,38-1,66 kod muškaraca.

**Zaključak:**

Formiran je panel RI za lokalno relevantne biohemijske parametre, ova studija je prva koja je sprovedena u našoj zemlji. Prilikom upoređivanja ovih rezultata sa drugim studijama i onima koje su prijavili proizvođači reagensa, uočene su neke razlike. Ova studija na taj način doprinosi povećanju pouzdanosti u tumačenju laboratorijskih rezultata, a samim tim i kliničkom vođenju pacijenata.

## Introduction

The clinical laboratory plays a crucial role in medical diagnosis by performing various types of tests on the same biological sample or in different sorts of samples. This helps prevent, forecast, diagnose and control the treatment of patients, making it an essential component of health systems with highly-trained personnel. In addition, the clinical laboratory ensures that accurate and timely results are provided through rigorous quality control [1]. The evolution of the clinical laboratory over the last few decades has meant a substantial change in the instrumentation used for analytical purposes, as well as in the requirements for obtaining, analysing and using the data, reinforcing its role in the field of public health. Such data are vital in understanding biological processes, their variability and the statistical bases that support them, which could significantly affect their interpretation [1].

The role of the clinical laboratory lies not only in assisting the diagnosis of a disease but also in the follow-up and epidemiological surveillance of this disease, allowing the evaluation of a series of parameters of genetic-metabolic diseases [2] [3], through the measurement of different laboratory markers (biochemical, haematological, etc.) that are essential for clinical decision-making and the selection of therapeutic strategies [4].

Each parameter reported by the clinical laboratory is accompanied by its reference value (referenceintervals; RI), which is a range of values of a measured quantity (measurand), which were obtainedfrom a group of reference individuals that have certain specific characteristics and that are commonly defined within the status of "healthy" individuals [5]. The values of these parameters must be analysed by the clinician together with the clinical history, anamnesis (signs and symptoms), and other features [6] [7]. Therefore, the RIs are an important factor in clinical decision-making as they directly influence the interpretation of the results thanks to their defined lower and upper limits, enabling the comparison of the results of a specific individual [8].

The variations in the results of different clinical laboratory analyses are related to intra-laboratory factors typical of the pre-analytical or analytical phases, where there may be variations in obtaining and handling samples, methodology, reagents and/or techniques used in the procedures. Extra-laboratory factors also play a role because involve the biological variations of individuals such as age, sex, nutritional, environmental factors and other co-variables such as ethnicity, which have been scarcely studied due to the complexity of obtaining representative populations of adequate size. The relevance of analysing ethnicity lies in the expected physiological and genetic and lifestyle variations that are expected to be found among the different populations around the world, which could significantly impact on RIs [8] [9] [10].

Clinical laboratories usually use the RIs recommended by the reagent manufacturers themselves,which are often obtained in other populations than those to which the assays are applied. However, the International Federation of Clinical Chemistry and Laboratory Medicine (IFCC) recommends using values that represent local populations [11], since, in some cases, the RIs of clinical tests show differences with respect to the intervals suggested by the manufacturers or even differ from the values suggested by international associations [9].

Latin American populations are the result of a mix of Caucasian, Amerindian, and Negroid populations. Recent studies have shown that the Chilean population has 42% Amerindian ancestry [12], especially in the northern and southern regions of the country. For this reason, based on the genetic background of the Chilean population, the establishment of local RIs is relevant. Previous studies in the Chilean population have focused mainly on obtaining RIs for adults in haematological parameters or in the hormonal study in paediatric groups [13] [14] [15]. Consequently, the objective of this study is to establish RIs for various biochemical markers of routine obtained from apparently healthy subjects of La Araucanía region in Southern Chile, treated at the Clinical Laboratory, Universidad Católica de Temuco.

## Materials and methods

### Study design

This is a non-experimental, retrospective study of univariate and multivariate analysis. Using an indirect »a posteriori« method, data was collected from 1,143 users. All parameters: glucose, urea, Aspartate aminotransferase (AST), Alanine aminotransferase (ALT), alkaline phosphatase, total bilirubin, direct bilirubin, uric acid, low-density lipoproteins (LDL), high-density lipoproteins (HDL), total cholesterol, low-density lipoproteins (VLDL), triglycerides, and total proteins (Table 1); were analysed using Cobas c311 analyser.

**Table 1:** Characteristics of biochemical marker tests (Roche Diagnostics).

Parameter	Method	Reference Interval<br>(Reported by<br>manufacturer)		Detection<br>limit	Linearity	Unit of<br>measurement	Interference
		Female	Male				
Glucose	Hexokinase	4.1–6.0		0.11	0.11–41.63	mmol/L	Jaundice,<br>Haemolysis,<br>Lipaemia
Urea	Kinetic test with urease<br>and glutamate<br>dehydrogenase.2	2.76–8.07		0.49	0.49 – 39.9	mmol/L	Jaundice,<br>Haemolysis,<br>Lipaemia
AST	IFCC / without peroxidal	≤0.53	≤0.67	0.08	0.08–11.67	μkat/L	Haemolysis,<br>Lipaemia
ALT	IFCC / without peroxidal	≤0.55	≤0.68	0.083	0.08–11.67	μkat/L	Haemolysis,<br>Lipaemia
Alkaline<br>phosphatase	Colorimetric assay in<br>accordance with a<br>standardised method	0.58–1.74	0.67–2.15	0.08	0.08–20	μkat/L	Haemolysis,<br>Lipaemia
Total Bilirubin	Colorimetric diazo<br>method	≤20.5	≤23.9	2.50	2.50–649.9	μmol/L	Haemolysis,<br>Lipaemia
Direct<br>Bilirubina	Diazo method	≤3.42		1.2	1.37–236.06	μmol/L	Haemolysis,<br>Lipaemia
Uric acid	Colorimetric enzymatic<br>test (Uricase/Peroxidase)	142–339	202–416	12	12–1487	μmol/L	Jaundice,<br>Haemolysis
LDL<br>Cholesterol	Enzymatic/Colorimetric	<2.59		0.1	0.1–14.17	mmol/L	Jaundice,<br>Haemolysis
HDL<br>Cholesterol	Direct<br>measurement/PEG	>1.68	>1.42	0.08	0.08–3.88	mmol/L	Jaundice,<br>Haemolysis,<br>Elevated<br>concentration<br>of fatty acids
VLDL<br>Cholesterol	Friedewald’s formula	–	–	–	–	–	–
Total<br>Cholesterol	CHOD–PAP	< 5.17		0.1	0.1 – 20.7	mmol/L	Jaundice,<br>Haemolysis
Triglycerides	GPO–PAP	< 2.26		0.1	0.1–10	mmol/L	Jaundice,<br>Haemolysis
Total Proteins	Biuret	64–83		20	20–120	g/L	Jaundice,<br>Haemolysis,<br>Lipaemia

Inclusion and exclusion criteria: Data from apparently healthy adult individuals from La Araucaníaregion, Chile, between 18 and 65 years old were collected from the database of the UC Temuco Clinical Laboratory at Universidad Católica de Temuco, Chile, who were assisted in the context of community health promotion and prevention campaigns.

Data located outside the limits of the analysis (as determined by Tukey’s Fences) was considered anoutlier and was excluded from further analysis. Data that were above the Clinical Decision Limits (CDL) or above the recommendations established for calculating RIs using mathematical models were excluded. Therefore, triglyceride values greater than 4.52 mmol/L were discarded for the calculation of LDL using the Friedewald equation [16] [17].

### Sample collection

Blood samples were obtained by venous puncture in the antecubital area using a vacuum system, via tubes with a clot accelerator and separator gel in individuals fasting for 10 to 12 hours. Subsequently, they were processed according to the recommendations of the UC Temuco Clinical Laboratory Manual. The serum was obtained by centrifugation at 2,500 rpm for 5 minutes, with no more than 20 minutes elapsing between extraction and centrifugation. Hemolysis, lipemia or jaundice were defined as criteria for rejection of the samples. The sera were analysed immediately after their collection without freezing cycles.

### Statistical analysis

Histograms were constructed for initial visual inspection and to assess the distribution of the data.The Kolmogorov-Smirnov test was used to verify the normality of the data. Non-parametric data were analysed with the Box-Cox method using Minitab version 19 statistical software. The Tukey Fence test was used to detect outliers, and for establishing the lower (Q1 - (1.5 x IQR)) and upper (Q3 + (1.5 x IQR)) limits, with IQR being the interquartile range (IQR = Q3 - Q1) [18]. The calculation of the RI was performed using the non-parametric indirect method based on interpercentile ranks recommended by the IFCC, a method that calculates the rank numbers of the 2.5 and 97.5 percentiles as Lower limit = 0.025 (n + 1) and Upper limit = 0.975 (n + 1), respectively [19]. Subsequently, the confidence interval of each percentile was determined using the binomial distribution [20]. To determine the differences in the sex variable, the Mann-Whitney test was used. Ratios (AST to Platelet Ratio Index (APRI index), Fibrosis-4 (FIB-4), and AST: ALT) were calculated using previously-published formulas [21]. All calculations were performed with GraphPad Prism version 8.0.1.

### Ethical Considerations

This project was approved by the accredited Research Ethics Committee of the Universidad Católica de Temuco (N° 011601/23) and carried out in compliance with the Medical Association Declaration of Helsinki Ethical Principles for Medical Research Involving Human Subjects.

## Results

This study included blood samples from 1,143 adult individuals (47.5% men and 52.5% women) with ages ranging from 18 to 65 years (mean age 38 years, median 34, and standard deviation 14). The results of the normality analysis using the Kolmogorov-Smirnov test are shown in Table 2. The parameters with normal distribution in men were urea, ALT, alkaline phosphatase and HDL, meanwhile in women were AST, ALT, total bilirubin, LDL, HDL, total cholesterol and triglycerides. In order to establish RIs via the non-parametric method, outliers were excluded by using visual inspection (histograms) together with the Tukey’s Fences method. Table II shows the total number of excluded data (outliers) and included data for estimating the RIs of each parameter, evidencing that the parameters (ratios) that present fewer atypical data were VLDL, FIB-4, AST/ALT, HDL, ALT, LDL, Total Proteins and the parameters with the most data excluded were glucose, AST, direct bilirubin and total cholesterol.

**Table 2:** Distribution of data by gender, outliers and the final number of samples used to estimate RIs. As determined by the Kolmogorov-Smirnov normality test<br>+ Values that do not meet normality, p <0.05<br>* Values that meet normality, p >0.05<br># Values obtained using the Friedewald equation

	Male			Female		
Parameter	Normality	Atypical<br>values	Final data<br>number	Normality	Atypical<br>values	Final data<br>number
Glucose	<0.0001^+^	43	492	0.0302^+^	16	584
Urea	0.0545^*^	10	526	0.0286^+^	10	590
AST	<0.0001^+^	23	502	>0.1000^*^	11	589
ALT	>0.1000^*^	6	518	>0.1000^*^	5	595
Alkaline phosphatase	0.0963^*^	7	527	0.0002+	15	585
Total Bilirubin	0.0337^+^	9	516	>0.1000^*^	9	591
Direct Bilirubin	0.0251^+^	7	514	<0.0001^+^	21	579
Uric acid	0.0005^+^	13	517	0.0012^+^	6	594
LDL Cholesterol	>0.1000^*^	6	501	>0.1000^*^	4	596
HDL Cholesterol	>0.1000^*^	5	538	>0.1000^*^	8	592
VLDL# Cholesterol	<0.0001^+^	0	511	<0.0001^+^	0	599
Total Cholesterol	<0.0001^+^	18	525	>0.1000^*^	7	593
Triglycerides	<0.0001^+^	20	511	>0.1000^*^	1	599
Total Proteins	0.0116^+^	6	502	0.0012^+^	2	598
AST/ALT	<0.0001^+^	4	519	<0.0001^+^	8	592
FIB-4	<0.0001^+^	0	523	<0.0001^+^	5	595
Apri index	<0.0001^+^	25	498	<0.0001^+^	16	584

The RIs obtained from the adult population (male and female) are presented in Table 3. The VLDL value was calculated according to the Friedewald equation [22]. Interestingly, all RIs differ between men and women, in particular, the parameters of urea, AST, ALT, total bilirubin, direct bilirubin, uric acid, LDL, VLDL, total cholesterol, triglycerides, and total protein, whose values were significantly higher in men compared to women. Conversely, the parameters of glucose and HDL were found higher in females than in males (p <0.05).

**Table 3:** Reference intervals calculated by sex. As determined by the Mann Whitney test for statistical significance<br>+ Values with significant differences between males and females, p <0.05<br># Values obtained with the Friedewald equation

Parameter	Unit of<br>measurement	Female		Male		Difference<br>female to<br>male<br>(P value*)
		Lower Limit	Upper Limit	Lower Limit	Upper Limit	
Glucose	mmol/L	4.58 (4.55–4.63)	6.64 (6.38–6.93)	4.77 (4.70–4.81)	6.45 (6.28–6.57)	<0.0001^+^
Urea	mmol/L	2.48 (2.28–2.54)	7.35 (7.02–7.79)	3.28 (3.16–3.36)	8.17 (7.94–8.37)	<0.0001^+^
AST	μkat/L	0.20 (0.19–0.20)	0.69 (0.63–0.83)	0.22 (0.20–0.23)	0.80 (0.78 -0.85)	<0.0001^+^
ALT	μkat/L	0.13 (0.12–0.15)	1.12 (0.96–1.46)	0.18 (0.01–0.2)	1.9 (1.8–2.25)	<0.0001^+^
Alkaline<br>Phosphatase	μkat/L	0.83 (0.82–0.87)	2.50 (2.35+2.62)	0.93 (0.87–0.97)	2.33 (2.28–2.40)	0.0002^+^
Total Bilirubin	μmol/L	3.52 (3.32–3.75)	17.39 (16.28–19.70)	4.58 (4.04–4.82)	24.08 (22.50–25.89)	<0.0001^+^
Direct Bilirubin	μmol/L	1.50 (1.37–1.61)	4.91 (4.72–5.13)	1.86 (1.76–1.98)	7.51 (6.86–7.85)	<0.0001^+^
Uric acid	μmol/L	148 (136–154)	362 (350–374)	202 (184–214)	458 (446–469)	<0.0001^+^
LDL Cholesterol	mmol/L	1.40 (1.31–1.46)	4.65 (4.60–4.87)	1.30 (1.17–1.48)	4.82 (4.69–5.16)	0.0136^+^
HDL Cholesterol	mmol/L	0.92 (0.86–0.96)	2.43 (2.33–2.54)	0.68 (0.65–0.72)	1.79 (1.75–1.84)	<0.0001^+^
VLDL Cholesterol^#^	mmol/L	0.24 (0.22–0.26)	1.54 (1.39–1.6)	0.26 (0.25–0.28)	1.88 (1.74–1.99)	<0.0001^+^
Total Cholesterol	mmol/L	3.18 (2.96–3.30)	6.83 (6.70–7.13)	3.4 (3.36–3.47)	7.27 (7.04–8.28)	0.0019^+^
Triglycerides	mmol/L	0.53 (0.47–0.57)	3.36 (3.02–3.49)	0.57 (0.54–0.62)	4.10 (3.79–4.34)	<0.0001^+^
Total Proteins	g/L	66.3 (65.7–67.1)	80.3 (79.6–81.1)	67.4 (66.5–68.4)	83 (82.6 – 88.3)	<0.0001^+^
AST/ALT	--	0.52 (0.49–0.55)	2.00 (1.90–2.09)	0.38 (0.36–0.41)	1.66 (1.46–1.74)	0.0004^+^
FIB-4	--	0.23 (0.21–0.25)	1.62 (1.40–1.87)	0.27 (0.25–0.29)	1.67 (1.55–1.79)	<0.0001^+^
APRI index	--	0.12 (0.11–0.13)	0.50 (0.47–0.57)	0.12 (0.11–0.13)	0.49 (0.48–0.50)	<0.0001^+*^

On the other hand, the IRs found in this work showed some differences compared to those IRs suggested by reagent manufacturers (Table 1 versus Table 3), especially for ALT and direct bilirubin.

Additionally, we have studied three biochemical ratios associated with liver damage and non-invasive evaluation of liver fibrosis (AST/ALT, FIB-4 and APRI INDEX). The FIB-4 index in our population presented a range of 0.2 to 1.6, the APRI index from 0.12 to 0.5, and the AST/ALT index was 0.5–2 in women and 0.4–1.7 in men (Table 3).

## Discussion

It is essential that each clinical laboratory determines, or at least validates, the RIs used in the population in which it provides services, considering both intrinsic and extrinsic variability [23]. In Latin America there are few studies that have established biochemical RIs [9] [24] [25] [26] [27] [28] [29] [30] [31], focusing on adults only in Mexico [25] and Perú [27], in Chile there are not publications in the area. Considering the heterogeneity of the Chilean population along the territory, this study aimed to establish RIs for various biochemical markers of routine use for the population of La Araucanía region in Southern Chile.

Table 4 shows the RIs obtained in this study compared to those found in other selected countries,including some countries from Latin America. In the case of the study performed in the Mexican population where the non-parametric method was used to analyse 653,467 clinical biochemistry data points, and the Tukey test was employed for the detection of outliers, greater differences were observed in the parameters of AST (in men) and ALT (in both sexes) compared to our study. Meanwhile, in the study performed in the Peruvian population, which used parametric statistics and Student's T-test to calculate outliers in 120 samples, a greater similarity is observed in the vast majority of parameters (including triglycerides), except in the case of uric acid [25] [27].

**Table 4:** Reference Intervals obtained in this study compared with those from selected other countries. NA, Not Available.<br>Values transformed according to the conversion of international units

Parameter	This study (Chile)		México [25]		Perú [27]		Ethiopia [32]		USA [33]	Tanzania [34]		Unit of<br>measurement
	Female	Male	Female	Male	Female	Male	Female	Male	Adults	Female	Male	
Glucose	4.58–6.64	4.77–6.45	NA	NA	3.95–7.89	3.72–5.88	3.33–6.61	3.61–6.99	4.16–6.38	3.30–5.06	2.88–5.30	mmol/L
Urea	2.48–7.35	3.28–8.17	2.13–6.83	2.50–7.16	3.00–7.16	2.50–7.16	1.33–6.91	2.16–7.84	NA	1.47–4.61	1.57–5.01	mmol/L
AST	0.20–0.69	0.22–0.80	0.20–0.58	0.20–0.58	NA	NA	0.267–0.986	0.318–1.05	0–0.58	0.225–0.586	0.253–0.89	μkat/L
ALT	0.13–1.12	0.18–1.9	0.116–0.60	0.157–0.783	NA	NA	0.132–0.80	0.234–0.816	0–0.58	0.117–0.748	0.153–0.921	μkat/L
Alkaline<br>phosphatase	0.83–2.5	0.93–2.33	NA	NA	NA	NA	0.833–5.432	0.916–5.72	0.5–2	0.72–2.585	0.757–2.83	μkat/L
Total<br>Bilirubin	3.52–17.39	4.58–24.08	3.76–17.78	3.76–17.7	NA	NA	1.71–20.52	1.71–22.2	5.13–17.1	4.5–31.3	6.0–42.0	μmol/L
Direct<br>Bilirubin	1.50–4.91	1.86–7.51	2.05–7.18	2.05–7.18	NA	NA	NA	NA	1.71–5.13	0.70–5.83	0.93–8.43	μmol/L
Uric acid	148.5–362	202–458	142–426	193–547	95–631	71–559	NA	NA	NA	148–360	196–459	μmol/L
LDL<br>Cholesterol	1.40–4.65	1.30–4.82	NA	NA	2.11–4.82	1.02–5.72	NA	NA	<2.59	NA	NA	mmol/L
HDL<br>Cholesterol	0.92–2.43	0.68–1.79	NA	NA	0.78–1.94	0.78–1.76	NA	NA	<1.03	NA	NA	mmol/L
VLDL<br>Cholesterol	0.24–1.54	0.26–1.88	NA	NA	0.25 – 1.42	0.2–1.63	NA	NA	NA	NA	NA	mmol/L
Total<br>Cholesterol	3.18–6.83	3.40–7.27	NA	NA	4.42–6.26	2.64–7.51	1.78–5.59	2.51–5.40	<5.17	2.82–5.5	2.32–5.67	mmol/L
Triglycerides	0.53–3.36	0.57–4.10	NA	NA	0.54–3.09	0.44–3.54	0.52–2.33	0.50–2.49	NA	0.38–2.18	0.39–3.01	mmol/L
Total<br>Proteins	66.3–80.3	67.4–83.0	62.0–81.0	65.0–81.0	56.0–82.0	60.0–83.0	52.0–90.0	58.0–85.0	62.0–82.0	65.8–85.5	67.2–85.2	g/L

When comparing our study with countries having different ethnicities (Table 4), we found that, in the Ethiopian population, where non-parametric statistics (direct method) were used in 344 users and Dixon's Q used for the detection of outliers, there was a greater difference in the IR values of parameters such as AST and alkaline phosphatase [32]. Similar results were reported in a review conducted in the United States, whose IRs values of AST, ALT and alkaline phosphatase showed more substantial differences compared to those found in our study [33]. Interestingly, our study showed IR values similar to those found in the Tanzanian population [34].

The values obtained in our study for glucose and lipid profile parameters are above international recommendations for clinical decision-making. For this reason, it is important to differentiate between RIs and CDLs; whilst RIs describe the typical (statistically normal) distribution of values observed in a healthy reference population, CDLs are associated with a significantly increased risk of adverse clinical events and may be associated with a specific disease [35]. Although the RI and the CDL values will be sometimes equivalent for some biochemical markers, this will not be always the case. For example, in the cases of the lipid profile, glucose or glycated haemoglobin, the RIs used are typically associated with the CDL because the values are determined more epidemiologically than statistically, following an international scientific consensus based on survival or incidence data obtained from studies where clinical complications were analysed according to the levels (below or above) from the established value (CDL or IR) [36]. This could explain why, in RI studies based on statistical approximations, the values found differ from those suggested by reagent manufacturers or from the CDL established by national or international health organisations.

The RIs for the lipid profile are based more on the CDL established by expert committees such as the Adult Treatment Panel III (ATP-III) using an epidemiological approach, rather than on studies of RIs based on statistics (Bayesian approach), as occurred in our study. This fact, together with the high levels of adiposity and risk factors for chronic diseases in the Chilean population [37], could result in a high probability that the RIs determined here were different from those previously reported. At the same time, this is an indirect indicator of the health status/risk factors of the population from which the reference individuals were obtained. Particularly for triglycerides, which is a parameter highly dependent on the diet and the time of year (circadian fluctuations), its variation among the Southern Chilean population may be associated with greater consumption of high-calorie products and sedentary lifestyles in the winter period, and obesity; all of them considered risk factors for non-communicable diseases such as cardiovascular disease, diabetes and dyslipidaemia [38]. These reasons may explain the higher values found in lipid profile parameters compared to values recommended by national bodies such as the Chilean Institute of Public Health (IPH). A similar situation occurs in the case of fasting glucose, where the American Diabetes Association recommends that the upper reference limit for this analyte is 5.55 mmol/L. However, the prevalence of undetected glucose disturbances is high (almost half of the patients with diabetes mellitus have not been diagnosed) in almost all populations of the world [39], increasing the possibility that the upper limit of RI is raised, even in apparently-healthy outpatients with impaired glucose metabolism. In addition, there are preanalytical factors (such as fasting), which are complex to analyse and control in RI studies with a posteriori selection of individuals.

The variations in these parameters may be also associated with individual factors such as age, tobacco use, fasting, and underlying diseases, among others [40] [41]. For all these reasons, it is recommended to use the reference values provided by health organisms in order to guarantee the correct diagnosis of pathologies associated with lipid and glucose metabolism [42] [43].

On the other hand, applying inappropriate statistical models to obtain IRS and inappropriate methodology for detecting outliers can significantly modify the results in this type of study [44]. In this work, we used an indirect method for the determination of RI for 14 biochemical parameters in the adult population. This method offers advantages over the traditional direct approach, since the use of pre-existing data in the database reduces the time, complexity, and cost of obtaining the results. In addition, this study used the same pre-analytical and analytical conditions that are commonly used in a clinical laboratory setting, also guaranteeing an adequate number of individuals from different ages, sex and ethnicities [45]. Some of the limitations of the indirect method are that not all the characteristics of the study population are known, which requires the use of appropriate statistical methods to exclude outliers. Likewise, this approach makes it difficult to incorporate additional inclusion/exclusion criteria or include other partition groups, such as pregnancy, lactation, neonates, ethnicity, priority pathologies in the region, diet, use of medications, physical activity, socioeconomic status, alcohol/tobacco consumption, etc. However, this indirect method is useful in local situations (small laboratories) or in specific populations (neonates, children or the elderly) being clinically relevant data for the laboratory that performs them, when a priori methods are not possible to implement [44].

Another relevant aspect of this work is the sample size, which included 1,143 data, which are well above IFCC recommendations (for non-parametric methods, the number of reference individuals must be at least 120 data) and, therefore, is more representative of the population. However, this sample size also generates a greater dispersion of the data in some of the parameters evaluated, which can be solved selecting the statistical tests according to the volume of data. On the other hand, all analytical variables were controlled according to the standards of a clinical laboratory accredited by the local health authority, and a detailed statistical analysis was performed to detect outliers.

Additionally, we studied three ratios associated with liver damage and non-invasive evaluation of liver fibrosis (FIB-4 index, APRI index and AST/ALT index) [46]. The FIB-4 index is obtained from the age, AST, ALT and platelet count, and it was originally developed to assess liver fibrosis in patients with HIV/HCV infection [24], with a cut-off value > 3.25 [47]. Recent reports have stated that FIB-4 index can be used in the diagnosis of different metabolic disorders, associated with a high risk of death from cardiovascular disease [48]. For instance, values of FIB-4 > 2.67 indicate a higher risk of hepatocellular carcinoma [49] and values of FIB-4 >1.10 suggest a higher risk of prevalent chronic kidney disease [50]. In the population of Southern Chile, we found values of of FIB-4 ranging between 0.2 and 1.6.

The APRI index is obtained from AST and platelet counts, and it is also used to assess liver fibrosis associated with hepatitis B or C [51], with a cut-off point of 0.5 for fibrosis and 1.5 for cirrhosis. APRI index can be also significantly correlated with cardiovascular risk when its value is > 0.5 (in both sexes) [52]. In our work, the APRI values were like those reported by other authors (0.12 to 0.5). Unlike other studies, Amernia et al. [53] found that APRI is the best index to predict advanced liver fibrosis compared to FIB-4 and the AST/ALT ratio, and can be also useful as a positive predictor of non-alcoholic fatty liver disease in severely obese children and adolescents. However, these authors conclude that APRI appears to be a simple biochemical marker of liver damage rather than a biomarker of non-alcoholic fatty liver disease [54].

On the other hand, the AST/ALT index obtained in this work was 0.5–2 in females and 0.4–1.7 in males, which is fairly similar to the figures reported by other authors, where most patients with heavy alcohol intake but without severe liver disease do not have an AST/ALT ratio greater than 1. A high AST/ALT ratio (usually > 2) suggests advanced alcoholic liver disease [55].

ALT has been widely used as a marker of liver damage [56] in transaminase-elevated alcoholic hepatitis with an AST/ALT ratio > 1.5, and with AST levels higher than 1.5 times the upper limit of normal [57]. In the Norwegian population, Haukeland et al. [58] reported that an elevated AST/ALT ratio was associated with increased mortality by alcoholic cirrhosis (AST/ALT > 2.42), being higher compared to non-alcoholic cirrhosis (AST/ALT > 1.42).

It is important to consider that the ratios that use ALT values such as AST/ALT and FIB-4 can be influenced by the increase in the activity of this enzyme. The ALT values obtained in our study showed an upper limit of 1.9 mkat/L in men, which may be associated with the high alcohol consumption among the adult and young population of La Araucanía region, according to the report by the National Service for the Prevention and Rehabilitation of Drug and Alcohol Con sumption (Senda) and other health organisms [59].

This is the first study that has established RIs for biochemical parameters and 3 ratios (FIB-4 index, APRI index and AST/ALT index) in adult individuals from La Araucanía Region in Southern Chile, observing interesting differences compared to those reported in other geographical areas of Latin America. Therefore, these data provide a valuable guide for clinical practice, and can serve as a reference source for future studies.

## Dodatak

### List of Abbreviations

RIs: Reference intervals;<br>IFCC: International Federation of Clinical Chemistry and Laboratory Medicine;<br>AST: Aspartate aminotransferase;<br>ALT: Alanine aminotransferase;<br>LDL: Lowdensity lipoproteins;<br>HDL: High-density lipoproteins;<br>VLDL: Low-density lipoproteins;<br>APRI index: AST to Platelet Ratio Index FIB-4: Fibrosis-4;<br>CDL: Clinical Decision Limits;<br>ATP-III: Adult Treatment Panel III;<br>IPH: Institute of Public Health

### Acknowledgements

The authors wish to express their appreciation to the work team at the Laboratorio Clínico UC Temuco. Research funding: Supported by VIPUCT-2022REG-PL-02 grant from the Vicerrectoría de Investigación y Postgrado, Universidad Católica de Temuco.

### Conflict of interest statement

All the authors declare that they have no conflict of interest in this work.
